# Medial septum: relevance for social memory

**DOI:** 10.3389/fncir.2022.965172

**Published:** 2022-08-23

**Authors:** Marilena Griguoli, Domenico Pimpinella

**Affiliations:** ^1^European Brain Research Institute (EBRI), Fondazione Rita Levi-Montalcini, Rome, Italy; ^2^Institute of Molecular Biology and Pathology of the National Council of Research (IBPM-CNR), Rome, Italy

**Keywords:** medial septum (MS), social memory, acetylcholine, hippocampus, CA2 region, neuropsychaitric disorders

## Abstract

Animal species are named social when they develop the capability of complex behaviors based on interactions with conspecifics that include communication, aggression, mating and parental behavior, crucial for well-being and survival. The underpinning of such complex behaviors is social memory, namely the capacity to discriminate between familiar and novel individuals. The Medial Septum (MS), a region localized in the basal forebrain, is part of the brain network involved in social memory formation. MS receives several cortical and subcortical synaptic and neuromodulatory inputs that make it an important hub in processing social information relevant for social memory. Particular attention is paid to synaptic inputs that control both the MS and the CA2 region of the hippocampus, one of the major MS output, that has been causally linked to social memory. In this review article, we will provide an overview of local and long range connectivity that allows MS to integrate and process social information. Furthermore, we will summarize previous strategies used to determine how MS controls social memory in different animal species. Finally, we will discuss the impact of an altered MS signaling on social memory in animal models and patients affected by neurodevelopmental and neurodegenerative disorders, including autism and Alzheimer’s Disease.

## Introduction

### Medial septum: “an information hub for social memory”

Establishing meaningful relationships among individuals is crucial for well-being and survival of social species. Social cognition includes a variety of complex behaviors that span from aggression and avoidance to cooperative and mating actions. All these functions rely on the capacity of an individual to recognize another, namely social memory, which is thought to be perturbed in various neuropsychiatric disorders, including schizophrenia, autism and neurodegenerative diseases.

Among different brain networks contributing to social memory formation, the medial septum-diagonal band of Broca complex (MS) has recently emerged as a relevant region (Pimpinella et al., 2021; Wu et al., 2021). The MS has been mainly studied for its bidirectional communication with the hippocampus and its contribution to hippocampal-dependent contextual memory (Hasselmo, 2006; Khakpai et al., 2013). In the last years cognitive functions associated to the hippocampus have been broadened toward social aspects, thereby the MS-hippocampal pathway has started to receive attention for its possible role in processing social information.

In rodents, social memory has been causally linked to the activity of the dorsal CA2 and the ventral CA1 hippocampal regions (Hitti and Siegelbaum, 2014; Okuyama et al., 2016). Recent evidence pointed out the requirement of the MS-CA2 pathway for social novelty discrimination and social memory stability (Pimpinella et al., 2021; Wu et al., 2021). In humans, the activation of neurons in the temporal lobe, including the hippocampus, has been associated to face recognition (Quiroga et al., 2005) but the role of MS in this process has not been elucidated yet.

The MS, localized in the basal forebrain, contains highly interconnected neurons releasing acetylcholine (ACh), γ-aminobutyric acid (GABA) and glutamate (Müller and Remy, 2018). Molecular markers for these neurons are choline acetyltransferase (ChAT), glutamate decarboxylase 67 (GAD67) and vesicular glutamate transporters 1, 2 and 3 (VGlut1 and VGlut2, VGlut3), respectively (Sotty et al., 2003; Gritti et al., 2006). ChAT-positive neurons are involved in aversive associative learning and spatial memory formation (Ikonen et al., 2002; Lovett-Barron et al., 2014). VGlut-positive neurons are activated in response to locomotion (Fuhrmann et al., 2015) and GAD67-positive cells are involved in memory consolidation (Boyce et al., 2016). A subpopulation of MS neurons co-expressing ChAT and GAD67 markers (Sotty et al., 2003) has the biochemical machinery to release both ACh and GABA in the targeted areas *via* co-transmission mechanisms (Desikan et al., 2018; Takács et al., 2018). Moreover, a fraction of ChAT- and GAD67-positive neurons can express VGlut2 and VGlut3 at axon terminals and somato-dendritic levels, respectively (Gritti et al., 2006). Thus, ChAT and GAD-expressing neurons have also the potential to synthesize glutamate and the vesicular transporters necessary for its release along with ACh or GABA as neurotransmitters. Remarkably glutamate binding to VGlut3, expressed at somato-dendritic level, may act as a retrograde signal upon afferent stimulation. How the signaling of the ACh and GABA/glutamate co-transmission is regulated and its functional implications are virtually unknown.

In addition to local connections, MS neurons integrate abundant cortical and subcortical inputs including those from the prefrontal cortex, the olfactory bulb, the lateral septum (LS). They establish bidirectional connections with several brain regions including the hippocampus, entorhinal cortex (EC), amygdala, hypothalamus, and brainstem. MS outputs ultimately form unidirectional synaptic contacts within the medial and lateral habenula, the mediodorsal nucleus of thalamus and cortical regions such as the pyriform and anterior cingulate cortex (Mocellin and Mikulovic, 2021; Takeuchi et al., 2021). Among multiple MS synaptic inputs, of particular interest are those originating in the supramammillary nucleus (SuM) of the hypothalamus, LS and EC, known to carry social information and to be relevant for CA2-mediated social memory processing (Dudek et al., 2016; Piskorowski and Chevaleyre, 2018).

SuM neurons are potential candidates for encoding the novelty features of social recognition as they preferentially activate in response to social novelty (Chen et al., 2020). SuM axon terminals form asymmetric glutamatergic synaptic contacts on parvalbumin-positive (PV) GABAergic interneurons and cholinergic neurons in the MS (Leranth and Kiss, 1996; Borhegyi et al., 1998). PV cells of the MS innervate hippocampal interneurons and contribute to theta generation by triggering disinhibition of principal cells (Tóth et al., 1993, 1997; Hangya et al., 2009). MS cholinergic neurons instead, provide a diffuse innervation of hippocampal neurons: they modulate theta power and inhibit sharp wave ripples (Lee et al., 1994; Vandecasteele et al., 2014; Dannenberg et al., 2015; Petersen and Buzsáki, 2020). By providing excitation to both MS interneurons and cholinergic cells, SuM inputs contribute to both theta generation and power in the hippocampus. Social stimuli increase the power of theta oscillations (Tendler and Wagner, 2015) while social dysfunction have been associated with a reduction (Modi et al., 2019). It is not completely known whether this involves hippocampal circuits and brain regions related to different types of memory (e.g., spatial).

Furthermore, Robert et al. (2021) demonstrated that SuM inputs drive PV-mediated feed-forward inhibition in CA2, thus securing a correct balance between excitation and inhibition. It is tempting to speculate that this circuit may have a role in social behavior, as alterations in PV-mediated transmission affect social memory (Piskorowski et al., 2016).

GABAergic and cholinergic neurons in the MS are likewise targeted by inhibitory projections from the LS (Leranth et al., 1992). LS is crucial for integrating social experience and internal states, thus shaping the socio-behavioral output including social interaction, aggression, sexual behavior and maternal behavior (Menon et al., 2021). Interestingly LS receives glutamatergic inputs from the CA2 region, whose activation precedes social aggression and it is controlled by vasopressin receptors expressed on CA2 axon terminals (Leroy et al., 2018). Therefore, the activation of LS by CA2 axon terminals in turn inhibits MS neurons. This inhibition may lead to theta reduction and SWR increase, favoring social memory reactivation (Hasselmo, 2006; Oliva et al., 2020).

MS establishes bidirectional connections with EC. Glutamatergic and, to a lesser extent, GABAergic projections from superficial layers of medial EC contact MS neurons (Fuchs et al., 2016). Cholinergic, glutamatergic and GABAergic inputs project in turn to the medial and lateral portions of the EC (Gonzalez-Sulser et al., 2014; Fuchs et al., 2016; Desikan et al., 2018; Viney et al., 2018). This loop may control the integration of sensory inputs relevant for social memory as the lateral part of the EC projecting to CA2 region has been recently shown to convey sensory information for this cognitive function (Lopez-Rojas et al., 2022).

**Figure 1:**
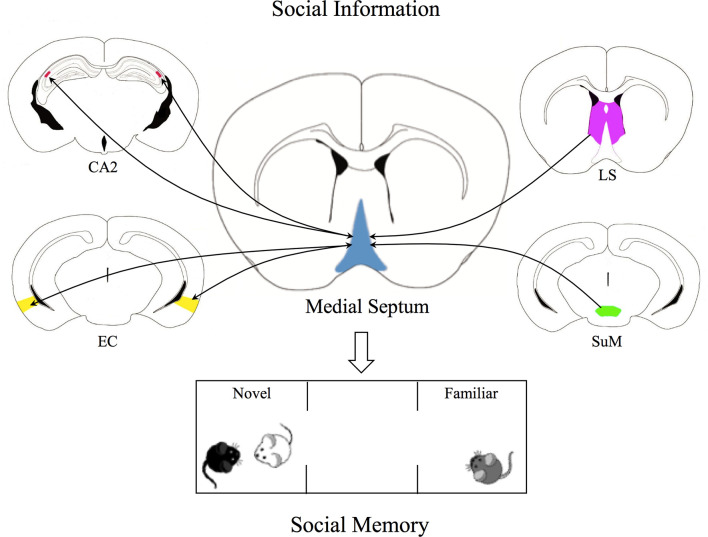
The Medial Septum as a social recognition hub. Graphical representation of the Medial Septum (MS) as an integration center of social inputs from supramammillary nucleus (SuM) of the hypothalamus, lateral septum (LS), and entorhinal cortex (EC). SuM neurons send glutamatergic projections to parvalbumin-positive GABAergic interneurons and cholinergic neurons in the MS, contributing to regulation of MS-CA2 axis, crucial for social memory. GABAergic and cholinergic neurons in the MS receive inhibitory projections from the LS which in turn receives excitatory inputs from CA2, thus these projections indirectly inhibit MS neurons activity. In addition, glutamatergic and GABAergic projections from medial EC establish synaptic contacts with MS neurons, while MS neurons project to the medial and lateral portions of the EC. These bidirectional connections may be crucial for the integration of sensory inputs relevant for social recognition. All these pieces of evidence shed light on the complexity of the role of MS as a social hub able to integrate social information and to regulate social memory.

The temporal dynamics underlying MS integration of “social” signals from SuM, LS, and EC inputs are completely unknown. Moreover, how MS computes this information and those coming from local connections and hippocampal afferents (Cui et al., 2013) has not been investigated yet. Although future work is needed to clarify this functionally important aspect, a clear picture representing the MS as a complex “hub” integrating social information relevant for social memory formation emerges (Figure 1).

### Medial septum: contribution to social memory

Diverse strategies including rather selective MS lesions, have been exploited to determine the role of MS in controlling social behavior. Broad MS lesions induced by the antimitotic drug vincristine impair social recognition of juvenile conspecifics in rats (Terranova et al., 1994). Moreover, selective MS cholinergic lesions induced by IgG2-saporin immunotoxin impair the retrieval of social transmission food preference, a social memory task that uses olfactory cues acquired before the toxin injection (Vale-Martínez et al., 2002).

Interestingly, in sheep cholinergic neurons in the basal forebrain including those localized in the MS are important for offspring recognition. The impaired lamb recognition is mainly due to an impairment in the acquisition of the offspring olfactory signatures and only partially to visual discrimination deficits (Ferreira et al., 2001). Olfaction-mediated recognition temporally precedes the occurrence of visual-auditory learning and these are independent processes associated to different amount of ACh release (Ferreira et al., 2000). ACh peaks within 2–4 h after parturition and abruptly decreases afterward (Kendrick et al., 1986; Lévy et al., 1993). This would explain the major impact of cholinergic damage on olfactory discrimination as compared to the visual-auditory ones.

Deficits in social memory observed in ewes treated with immunotoxin do not depend on an altered maternal care (Ferreira et al., 2001) as supported by the evidence that scopolamine administration does not affect it (Lévy et al., 1997). Furthermore, olfactory function seems intact as lesioned ewes respond correctly to attractive or repellant odors (Lévy et al., 1983; Ferreira et al., 2001), excluding alterations in sensory processing. It is worth to mention that social recognition relies on the integration of complex neural mechanisms including the oxytocinergic signaling (Ferguson et al., 2000) whose interaction with cholinergic ones during lamb recognition learning (Lévy et al., 1995) may tune circuit specificity for social memory.

In adult mice, cholinergic signaling is crucial for social memory as well, as cholinergic neurons activate in response to social stimuli. In addition, the selective silencing of MS cholinergic neurons by tetanus toxin or chemogenetic approaches impairs the recognition of a novel mouse over a familiar one in the three chamber test (Pimpinella et al., 2021). These manipulations do not affect the preference for an animal as compared to an object indicating that MS signaling is not required for social interactions (Pimpinella et al., 2021; Wu et al., 2021; but see Okada et al., 2021). Social memory is under the control of ACh released in the CA2 region of the hippocampus as cholinergic axon terminal inhibition or nicotinic ACh receptors (nAChRs) blockade mimic MS cholinergic neuron silencing (Pimpinella et al., 2021). Here, ACh preferentially targets nAChRs expressed on GABAergic interneurons leading to principal cell disinhibition (Pimpinella et al., 2021). Wu et al. (2021) highlighted the critical role of MS in controlling social memory encoding but not recall by promoting synaptic plasticity in the dorsal CA2 region; this occurs *via* 5-HT_1B_ receptor activation in the MS by serotonin release from median raphe axon terminals. All these pieces of evidence suggest that complex interactions among different neuromodulatory and neurotransmitter systems across several brain networks underlie social memory.

### Medial septal dysfunction in neuropsychiatric disorders

The role of the MS in regulating social behavior in mammals has only recently emerged, paving the way for the development of new therapeutic strategies aimed at curing a variety of neurodevelopmental and neurodegenerative diseases in which these social skills are impaired, including Autism Spectrum Disorders (ASDs) and Alzheimer’s disease (AD).

#### Autism spectrum disorders

ASDs are neurodevelopmental disorders characterized by reduced social interactions, communication deficits, and stereotyped behaviors. Autistic individuals show substantial social impairments, having a difficult time interacting with others and paying attention to social stimuli.

Neuroanatomical analysis of postmortem brain tissue and imaging studies in ASD patients have revealed alterations in neurons within the basal forebrain. In particular, autistic patients show smaller neurons and increased cell density in the MS as compared to age-matched healthy individuals (Bauman and Kemper, 2005). Among the large variety of ASD cases, patients affected by Rett (RTT) syndrome, as well as RTT animal models, show MS abnormalities. RTT is a syndromic form of ASD, associated in 95% of cases with mutations in the Mecp2 X-linked gene (Moretti and Zoghbi, 2006). In mice, the loss of Mecp2 from glutamatergic and GABAergic neurons in the basal forebrain recapitulates autistic-like behaviors. In particular, the lack of MeCP2 from excitatory neurons in the basal forebrain has been shown to cause cortical hyperexcitation and repeated seizures in mice, mimicking recurrent symptoms of RTT patients (Zhang et al., 2014). Furthermore, it was shown that the constitutive Mecp2 loss in a subset of forebrain GABAergic neurons is sufficient to cause repetitive behaviors, to impair motor coordination and to alter social interest in mice (Mecp2-/y mice; Chao et al., 2010). The expression of MeCP2 in the forebrain is crucial during development as the postnatal loss of MeCP2 in the forebrain induces abnormal motor coordination, increases the anxiety-like behaviors and reduces social interactions. All these behavioral abnormalities are similar to those observed in RTT patients (Gemelli et al., 2006). Interestingly, the conditional loss of MeCP2 in cholinergic neurons causes RTT-like symptoms in mice (ChAT-Mecp2-/y) and the selective MeCP2 reactivation in MS cholinergic neurons in ChAT-Mecp2-/y mice can reverse behavioral deficits. ChAT-Mecp2-/y mice exhibit social interaction deficits similar to those observed in mice carrying *Mecp2* deletion in glutamatergic and GABAergic neurons but a reduced anxiety-like behavior. In addition, ChAT-Mecp2-/y mice show social memory deficits that seem exclusively related to cholinergic dysfunction (Zhang et al., 2016). The altered phenotype observed in ChAT-Mecp2-/y mice has been linked to a deficit in the signaling pathway of α7 nicotinic ACh receptor subtype, activated by ACh released by MS cholinergic afferents in the hippocampus. This evidence confirms the relevance of the septo-hippocampal cholinergic pathway in social memory processing, as shown by Pimpinella et al. (2021), and highlights its possible role in the pathophysiology of Rett syndrome. These findings pave the way for future investigations on MS as a potential target region for treating social memory deficits in ASDs.

#### Alzheimer’s disease

AD is the most prevalent form of dementia characterized by beta-amyloid plaques and neurofibrillary tangles, which leads to memory loss and other cognitive impairments that interfere with everyday living. In AD, social recognition, which in humans may be evaluated as the capacity to identify familiar faces, is compromised (Wilson et al., 1982). Several AD mouse models show social deficits that are comparable to those observed in humans. Specifically, these models exhibit social memory deficits despite they display adequate social interaction (Faizi et al., 2012; Coutellier et al., 2014). Among the brain areas affected in AD and involved in social memory, the basal forebrain has received little attention in recent years. Poppe et al. (2019) reported that postnatal genetic ablation of the Ephrin receptor 4 in the forebrain ameliorates social memory in APP-PS1 mice, in association with rescue of beta-amyloid-induced dendritic spine loss (Poppe et al., 2019).

The cholinergic system of the human basal forebrain degenerates early during the progression of AD, possibly due to the sensitivity of cholinergic neurons to tau pathology, and asymptomatic patients also display cholinergic denervation (Cantero et al., 2020). Acetylcholinesterase inhibitors as Donepezil are among the most common authorized drugs for the treatment of AD. AD patients treated with Donepezil show an improvement in interpersonal relationships, social interactions and conversational skills as assessed by caregivers (Boada-Rovira et al., 2004). Furthermore, 1-year lasting Donepezil administration prevents cholinergic neuron degeneration in the basal forebrain and increases the density of cholinergic projections in the hippocampus (Cavedo et al., 2017). Moreover, in AD mouse models the dysfunction of cholinergic system within the MS is accompanied by neurodegeneration of GABAergic neurons (Loreth et al., 2012), pointing the attention on an altered whole septo-hippocampal axis in this pathology.

Overall this evidence point to the MS as a potential target for early diagnosis and treatment of AD. Considering the current findings on the relevance of the MS-hippocampal pathway for social memory, it will be particularly important to get insights into molecular fingerprints of this circuit that may be exploited for treatment purposes.

## Conclusions and Future Directions

MS is one of the most connected regions of the brain and recent evidence suggests that it takes part in the “social brain” network. Previous evidence indicates that neurons in the MS are engaged in social memory, meaning the capacity of an individual to recognize a previously encountered conspecific. Studies using opto-chemogenenetic tools to control selected MS neuronal populations have allowed clarifying the key role of MS in controlling social memory. However, how MS neurons integrate synaptic inputs from SuM, LS, and EC carrying social information relevant for memory encoding is virtually unknown. SuM and LS inputs drive excitation and inhibition to both cholinergic and GABAergic neurons in the MS, respectively. Hence, the net effect on hippocampal theta oscillations and sharp wave ripples, known to be controlled by MS, would be opposite and possibly involve different phases of social memory (e.g., encoding and reactivation). EC inputs trigger both excitation and, to a minor extent, inhibition in the MS that in turn controls EC activity *via* disinhibition. This loop may filter distinctive types of information carried by EC (e.g., spatial and social) and differently affect network activity in MS targeted regions. This complexity is increased by the fact that MS neuron activity is modulated by non-canonical neurotransmitters including dopamine, serotonin and noradrenaline, released by axon terminals originating in the Ventral Tegmental Area, Dorsal Raphe Nucleus and Nucleus Coeruleus, respectively. These neuromodulators can act on a longer time scale as compared to classical glutamatergic and GABAergic neurotrasmitters, thus influencing MS integration time window, output and internal brain states leading to an extensive flexibility in social behavior outcome. Thereby, future investigations are needed to determine how such different neuromodulatory systems interact in controlling incoming GABAergic and glutamatergic inputs and the rules of synaptic plasticity within MS local circuit and its target areas. A new generation of sensors that show an unprecedented spatial and temporal resolution combined with advancements in imaging methods that allow to investigate the activity of deep brain structures will help in dissecting out the contribution of single neuromodulators during behavioral tasks assessing social memory.

## Author Contributions

MG and DP wrote the manuscript and DP generated the figure. All authors contributed to the article and approved the submitted version.

## Funding

This work was supported by Fondo Ordinario Enti (FOE D.M 865/2019) funds in the framework of a collaboration agreement between the Italian National Research Council and EBRI (2019–2021).
